# Participant Anonymity in the Internet Age: From Theory to Practice

**DOI:** 10.1080/14780887.2014.948697

**Published:** 2015-01-21

**Authors:** Benjamin Saunders, Jenny Kitzinger, Celia Kitzinger

**Affiliations:** ^a^ Keele University, Arthritis Research UK Primary Care Centre, Research Institute for Primary Care and Health Sciences, Keele, UK; ^b^ Cardiff University, Chronic Disorders of Consciousness Research Centre, Cardiff School of Journalism, Media and Cultural Studies, Cardiff, Wales, UK; ^c^ University of York, Chronic Disorders of Consciousness Research Centre, Department of Sociology, York, UK

**Keywords:** anonymity, coma, Internet/online, research ethics, serious brain injury

## Abstract

Qualitative researchers attempting to protect the identities of their research participants now face a multitude of new challenges due to the wealth of information once considered private but now readily accessible online. We will draw on our research with family members of people with severe brain injury to discuss these challenges in relation to three areas: participant engagement with the mass media, the availability of court transcripts online, and participants’ use of social media. We suggest strategies for managing these challenges via disguise, refining informed consent, and discussion with interviewees. In the context of a largely theoretical literature on anonymization, this article offers concrete examples of the dilemmas we faced and will be of illustrative use to other researchers confronting similar challenges.

## Introduction

Does the Internet effectively mean the death of research participant anonymity? What can researchers do to try to protect the identity of those who participate in their research in light of the vast amount of highly personal and often detailed information available online, and the speed with which search engines allow for its retrieval and organization? Newspaper coverage is no longer ephemeral but leaves an indefinite Internet imprint; anyone can now access online transcripts of court hearings and coroners’ reports, and there is also the vast amount of information shared on social media platforms. All of this means that in many cases it is no longer necessary to know people’s names to assemble a rich profile of their lives and thereby identify their names via jigsaw identification.

So has it become impossible to effectively isolate the private information (conveyed in an interview under guarantees that research participants’ real names will not be used) from public information out there for anyone to see (e.g., a media report)? If it has, how should researchers respond? In this article we address these questions through offering concrete examples of the challenges we faced in anonymizing our data in the particularly sensitive area of severe brain injury. It is hoped that this discussion will be of help to other researchers confronting similar challenges.

### Ethical Dilemmas in Online Research

There has been a great deal of recent work looking into online research. Writers have discussed, among other things, the opportunities and challenges for research via the Internet (for a review, see Whitehead 2007), the use of the net as a medium to conduct interviews (Maczewski, Storey & Hoskins 2004), and the way users deploy the potential for anonymity online (e.g., Bargh & McKenna 2003). A significant body of work has focused on the ethical dilemmas in the use of online data, with scholars consistently asking questions such as: Should online data be considered public and thus fair game, or do those writing online have a right not to expect their words to be republished? When and how can informed consent be gained? And do online identities have the same right to confidentiality as those offline (Frankel & Siang 1999)? A report by Ess and the Association of Internet Researchers (AoIR) (2002) recommends that those working with online data adopt a middle ground approach in addressing these questions—not “anything goes” but also not a set of prescriptive rules that fail to account for contextual factors related to the data source. More recently, Markham and Buchanan along with the AoIR (2012) extend these recommendations in advocating a casuistic or case-by-case approach in which ethical considerations should be tackled as they arise throughout the research process.

In addressing some of these ethical issues, Sveningsson (2004) argues that online pseudonyms should be anonymized; although a person’s real name is hidden, he or she will likely retain the same online name which may be recognized by other online users. She highlights the need to consider the focus of the research in making ethical decisions, for instance, researching the content of people’s messages in order to assess personality types is more sensitive, and perhaps in need of a more carefully considered approach to informed consent, than looking into general patterns of online language use (2004).

The private versus public status of online data is further discussed by McKee and Porter (2009), who found that some spaces in online roleplaying games such as *Second Life* are considered more public or more private than others, which may affect when informed consent is deemed necessary in the use of this data; thus, these ethical issues must be tackled *within*, as well as across different sites of online communication. This is something similarly observed by Sveningsson Elm (2009), who problematizes the public/private dichotomy, arguing instead that online spaces should be considered along a continuum of public to private, reflecting the grey areas in between the two.

Social media has been a particularly rich topic for the discussion of online research, with some commentators arguing that online self-disclosure reflects a “culture of increasing individualisation where people want to have their story told” (Wiles et al. 2008, p. 426) and thus have less concern about privacy. Van den Hoonaard and van den Hoonaard (2013) propose that there is a “fair expectation” on the part of people who post “publicly available nonintrusive materials” online that “issues of privacy, confidentiality and anonymity have no currency” (p. 63). Though van den Hoonaard and van den Hoonaard (2013) fail to fully acknowledge the subjective nature of what may be deemed nonintrusive, they nevertheless recommend that even where information is considered public, researchers should maintain stringent ethical codes in the use of such data.

The idea that there is a contemporary apathy toward privacy is taken a step further in Senft’s (2008) use of the phrase “micro-celebrity” to refer to how some people attempt to overtly publicize themselves online, viewing their audience as a fan-base, and “‘amping up’ their popularity … using techniques like video, blogs, and social networking sites” (p. 25).

In contrast to this view, other commentators have argued that many people do in fact still hold expectations of privacy in relation to their online postings. For example, boyd and Marwick (2011) contend that the self-information teens post on Facebook shows them not disregarding privacy but negotiating it in different ways and “developing strategies for managing privacy” (2011, p. 26). They highlight the situational nature of privacy, drawing on Nissenbaum’s (2004) notion of “contextual integrity,” which asserts that the given contextual environment determines what is appropriate to ask and what should remain private and how information should be distributed. boyd and Marwick give an example of teens being upset when images from their Facebook pages were displayed at school by teachers and police in a talk about the dangers of posting information online. The teens saw this as a breach of their privacy as they had lost control of *how* these images were used; in their eyes accessibility had been wrongly conflated with publicity (2004).

Markham (2012, p. 3) similarly argues that “people may operate in public spaces but maintain strong expectations of privacy.” She too advocates a case-by-case approach in making ethical decisions about the use of online data, but she makes the point that assessing issues of privacy vis-à-vis a particular context at a particular point in time cannot account for future harm that may be encountered, as “what may seem ephemeral or innocuous at one point in time might shift rapidly into something that causes real or perceived harm” (2012, p. 4).

We have highlighted the wealth of literature on Internet research, which provides an important backdrop to the focus of this article and informs our approach to considering the public/private nature of online information. The majority of this work, however, has explored issues related to Internet data; there is relatively little research examining the implications of the World Wide Web for researchers using traditional face-to-face qualitative data-collection techniques and seeking to maintain participants’ anonymity. That is the gap we address here.

### Severe Brain Injury

We highlight the challenge of anonymizing conventional interview data in the age of the Internet by drawing on examples of the dilemmas we faced in seeking to protect the identities of our research participants: people who have—or had—a severely brain-injured relative in a vegetative state (VS) or minimally conscious state (MCS) (for more information about this data set, see Kitzinger & Kitzinger 2014). Our interviewees had painful and unusual stories to tell, and they sometimes told them not just to the interviewers in our research study but also to journalists, in courtrooms, and/or via social media. In publishing our research we needed to think about this whole network of information and the multitude of ways in which this could threaten anonymity, especially when research participants had confided parts of their story to the interviewer which they would not want publicly identified with their own name. Sensitive information included views about how other members of the same family had responded to the brain injury (something they might not have openly discussed within the family), statements about the clinicians treating the patient (never shared with the clinician for fear of threatening the patient’s treatment), and highly personal details of their relationship with the injured individual. Interviewees also clearly identified some things they spoke about as difficult to discuss, even with other families in the same situation, including, for example, the not uncommon belief among some of those we interviewed that their relative would not want to be kept alive in their current state and, in some cases the fact that the interviewee had considered “mercy killing” (Kitzinger & Kitzinger 2013). Research has a vital role to play in allowing such situations and responses to be analyzed and discussed—and anonymity remains an important consideration even in a world in which almost everything is accessible online because, as noted above, this accessibility should not necessarily be conflated with publicity (boyd & Marwick 2011).

In the sections that follow we map out the challenge posed by the easy retrieval of media reporting, court documents, and social media before reflecting on what the researcher can do to try to maximize anonymity in the age of the Internet.

## Media Reporting

In our data set, challenges to anonymity were especially potent in cases where interviewees’ stories had received widespread media attention. This might be because of media interest in a crime (e.g., an assault) resulting in severe brain injury, in a campaign or fund-raising activity for rehabilitation facilities, or in a court judgment (e.g., related to the continuation or discontinuation of life-sustaining treatment). Several commentators have made mention of how media attention can threaten anonymity (e.g., Kelly 2009), but this commonly refers to the effect of study findings in sparking media interest. In our own research, the concern (in some cases) was not that our participants would be identified in the mass media because of our research but rather that they may be identifiable in the research because they had already appeared in the media. A few of the families interviewed had, for instance, been involved in court cases regarding application for the withdrawal of artificial nutrition and hydration (ANH) from their relative. In some instances, not only had the story been reported in the media, but family members had also given newspaper interviews. The strong possibility that key quotations in our interviews may be similar or even identical to quotations used in media interviews could render a research participant identifiable. For example, a reader keen to uncover a participant’s identity could copy and paste key phrases from quotations in publications into an Internet search engine and might come up with media interviews. In other words, we were faced with trying to protect the identities of participants who have already made (part of) their stories publicly available in another format.

Discussion in the research literature about the public/private status of online information does not consider media coverage or interviews research participants may have given to journalists. By talking to the mass media these individuals were quite clearly intending to “go public” in a way that they may not be by writing on Facebook, for instance, but the publicity being sought here is very different from that discussed in the literature, such as in the case of Senft’s (2008) micro-celebrities. Interviewees in our research who had given mass media interviews mostly simply wanted a platform to tell their story in order to raise awareness of serious brain injury in general or the condition (and needs) of their relative in particular. In spite of their choice to go public, however, we came to the decision that it was essential for us to anonymize these participants in the same way as those who had not engaged with the mass media. The prior choice of some participants to speak publicly does not obviate the need to maintain participant anonymity as far as we can—especially as, in this research, the interviews were conducted by two insider researchers (with their own experience of having a family member with catastrophic acquired brain injuries), which meant that interviewees often talked in very different ways than they would have to a journalist (Kitzinger & Kitzinger 2014). As Kelly (2009) quite rightly argues (in relation to schools being televised, though the point is a general one), when people engage with the media, they are “*controlling [their] own* anonymity (or lack of it)” (2009, p. 442, emphasis in original). Although people may choose to identify themselves in one context, they also may wish for anonymity in another, where they may perhaps reveal different or additional information about their experience. We therefore took significant steps to anonymize these participants as far as we could; the strategies we used will be discussed below.

## Court Cases

When interviewees had been involved in high profile court cases, then—in addition to media reports—transcripts of court hearings may also be available online. Our data set includes families who had been involved in court cases prior to being interviewed, but we also did some interviews with families who were *subsequently* involved in court hearings—after our interviews had been anonymized and shared with the research team and after some quoted material had already been published. At the time when we were carrying out the anonymization we could not predict which information would later be made public, and therefore which details could threaten the anonymity of our participants. Court transcripts can contain substantial detailed information about patients and their families. In one recent case (*W v M & Ors* 2011), England and Wales High Court (EWHC) 2443 (COP) concerning a patient in MCS, the transcript of the court hearings contains the following details:

- She was 52 years old and had been with her partner since 1983.
- She worked as a hairdresser.
- Her brain injury resulted from viral encephalitis the day before she was due to go on a skiing holiday in 2003.
- She had been a fiercely independent and vigorous person.
- Everyone in her family supported treatment withdrawal to allow her to die.

This court transcript, like many in the Court of Protection that deal with the affairs of vulnerable adults who have lost capacity, is anonymous; the patient is referred to only as “M.” Furthermore, since the family of M were very concerned about press intrusion in their lives and considered whether or not to continue with the case once they discovered the press interest in identifying and contacting them, this case was the subject of a “superinjunction.” The superinjunction barred reporters from approaching 65 people linked to the case (the family, health care professionals, and others) and from going within 50 meters of M’s care home without permission. It was also the first injunction ever in the United Kingdom specifically banning publication of information about the case on Facebook or Twitter. Despite all of these precautions, however, the details contained in the court transcript could lead to the identification of the family if we had interviewed a family member and retained in the final anonymized version the details given above (and others like them). Once a court transcript and associated media reports have already appeared, we can anonymize our interview data accordingly; however, if an interview is conducted beforehand, details like the fact that the patient had worked as a hairdresser or run the London marathon would have seemed innocuous and only subsequent to publication of the court transcript would they have had the potential to identify the family. Cases like these presented challenges even after we had undertaken substantial anonymizing work (Saunders, Kitzinger & Kitzinger 2014), for example, substituting names and places with pseudonyms, carefully managing identifying details, and often changing nonessential information.

## Social Networking

Several participants used social media for fundraising (e.g., for a care home through sites such as Just Giving), and others used YouTube videos, Facebook, Twitter, and online blogs to share the experience of having a relative in a vegetative or minimally conscious state, to keep distant family and friends in touch with the patient’s condition and/or as a platform to raise awareness or to campaign for a political and legal change. This is not uncommon among families facing this situation; see, for example, the mass of information about brain-injured patient Ryan Diviney on the website set up by his family, http://ryansrally.org.

As highlighted above, in many cases families may not see themselves as “going public” via social networking in the same way as those who have given interviews to the mass media or been involved in court hearings and may still hold expectations of privacy in relation to the information they post (Markham 2012). Nevertheless, this information can pose similar risks in that particular quotes or information on these sites could mirror that given in the research interview. The risk of cross-linking this information is increased since the accessibility of online material often extends beyond posting information in the first instance; it is commonplace for information to be reposted, retweeted, and shared in various ways and thus be accessed by a much wider audience. Markham (2013) refers to this as the “remix and redistribut[ion] of data” (p. 288) that can see “information develop a social life of its own, beyond one’s immediate circumstances” (p. 290).

Whilst some participants using social media to talk about their family member may still desire anonymity, we found that those campaigning or trying to raise awareness were often, and understandably, keen to be identified. Some in fact expressed an explicit desire to be named in the research, often driven by an additional desire to honor the story of their brain-injured relative (see also Grinyer 2002). While some researchers have proposed embracing the potential for cross-linking research findings with online information as a way of empowering participants, for example, through “transcripts being published … on participants’ websites, on a common project website or the researcher’s website” (Maczewski et al. 2004, p. 73), this was something we actively avoided for the research as a whole, given the sensitivity of our research area. What is more, our ethical duty was not just to our research participants but also to any other individuals connected to their stories (including treating clinicians and, of course, the patient who could not consent on their own behalf or be consulted about their story being told). With this in mind, we took significant steps to anonymize these participants, as well as at times negotiating with participants about how to deal with parts of interviews which mirrored information publicly available online. However, this strategy was combined with initiating a *subsequent* project (funded by an ESRC knowledge exchange initiative) in which some family members were filmed—and clips from these interviews used, often alongside their real names, on a website which summarized the cumulative research findings and presented them in an accessible website to support other families, and provide training materials for practitioners (see healthtalkonline.org.uk). This project followed an entirely different protocol, with family members waiving some elements of anonymity while maintaining others (ESRC award [ES/K00560X/1]). One deliberate end point here was to work with families to create more accessible and engaging materials than simple written quotations. It also served to challenge the invisibility (and often shame) attached to some aspects of experience. For example we filmed families who had supported applications to court to have their relative’s feeding tube withdrawn. These families had often been advised (or compelled by the court) to keep this secret—and in one case had even disguised what was going on from other members of the family.

## What Can Researchers Do?

We have so far outlined the challenges that online information posed to our attempts to anonymize our interviews; however, we are not suggesting as a result that anonymizing qualitative data is a fruitless task. In spite of the difficulties faced, we were able to devise strategies that minimized the risk of participant identification, although in order to describe these here we will sometimes have to use invented examples so as not to risk breaching the very confidentiality our strategies are designed to protect. In addition to the usual strategies of changing names, place names, occupation, and taking care with other potentially identifying details such as culture and religion (a far from straightforward process, as discussed in Saunders et al. 2014) we also at times developed more elaborate disguises involving changing key (but nonessential) elements of an account. Suppose, for example, that an interviewee had told us that it was horrific to consider ANH withdrawal from her daughter in a permanent vegetative state because that daughter had suffered from anorexia—and further suppose that this case had come to court, and a transcript was available on the Internet in which the mother referred to her daughter’s anorexia. This is sufficiently unusual (in fact, there is no such case) that it would identify not only the mother and daughter but also other members of the family and any health professionals mentioned in the interview. In anonymizing this interview for publication we would have changed the daughter’s illness from anorexia to, for instance, oesophageal cancer—another illness (albeit physical rather than psychological) that would have caused problems in eating and might therefore similarly affect how the mother felt about ANH withdrawal. If we think that disguise may be too transparent, the daughter may become a son, and/or the mother transformed into a sister. In this way, we altered certain details, while taking care not to affect the essence of the story. This could be seen to overlap with Markham’s (2012) discussion of the “fabrication” of data, a method she endorses as a way of protecting participants’ identities. However, at no point did we create representational accounts which do not use the actual words spoken by participants, one method Markham discusses; thus, all of our data originated from the participants themselves. Additionally, while Markham talks of creating composite accounts, that is, putting together extracts from different individuals to make a whole representational account, we more commonly did the opposite, that is, presenting extracts from the same participant under multiple pseudonyms, a strategy also discussed by others (e.g., Kaiser 2009; van den Hoonaard & van den Hoonaard 2013). While it could be argued that this may mislead readers, we saw this as a necessary measure as linking several different extracts to the same individual would greatly increase the risk of identification.

Through this anonymizing strategy we attempted to strike a balance between protecting participants’ identities as much as possible while still maintaining the integrity of the data to as great an extent as possible. For further discussion, see Saunders et al. (2014) and others who have discussed practical anonymizing methods (e.g., Clark 2006; Clough & Conigrave 2008).

Further attempts to address anonymizing issues were made through collaboration with interviewees and reflecting with them on how important anonymity could be for them and whether some of the material they shared with us was more sensitive than others. We found a range of views among interviewees about the level of protection they wanted. Some interviewees felt we were being overprotective and even resisted efforts to ensure their anonymity—this could include, for example, an express wish that we use not only their real name but also the real name of the patient (something we could not usually do as the patient was unable to give consent). Other interviewees wanted to maximize anonymity, including, for example, ensuring that some of what they said would not be recognized by other family members.

In some cases we discussed the problem of anonymizing their stories not only in advance of but also during the course of the interviews. For example, one interviewee who described an occasion when he felt close to killing his relative had originally accepted with equanimity that, since his story had already been reported publicly in the media, it might be possible to identify him from his research interview. However, when the interviewer revisited this issue during the course of the interview and suggested that the section of the interview where he describes an incident when he took rubber gloves into the care home in anticipation of committing murder, go in under a different pseudonym, he rapidly concurred: that part of the interview was not something that he had, or would, tell a journalist.

We also tried to be very explicit with interviewees about the extent of anonymity we could offer, and its limits, and to spell this out not only on paper in advance, but also to underline the issue at the end of the interview in person. For example the following statement was made to an interviewee toward the end of the interview:

We will change the names—your name and the names of people in your family and the names of any professionals. We’ll change the name of any hospitals and residential care homes or hospices. We will modify your occupation if you mention it and that of the brain-injured person. And if you have any specific requests, we’ll alter other details: for example, we can split to use different pseudonyms, as we’ve just discussed. So if it’s about the court case explicitly, that will have one pseudonym and that will separate it out. But when people blog and everything else, we can’t guarantee nobody will make a link between an interview with us and your going public in a blog, or Facebook or to a journalist.

We also sometimes revisited anonymity issues with interviewees some time after the interview with those who stayed in touch with the project. While some welcomed this, others (such as one whose relative had died and was trying to move forward) felt they had given the interview, which had often opened up painful memories, and they did not necessarily want to keep revisiting the material. They wanted simply to trust the researchers to make good use of the material provided to help other families and keep in touch to see how the work was developing.

As we became aware of the problems we were encountering, we also resubmitted our participant informed consent form to our university ethics committee with an amendment, as shown in Table 1.

**Table 1:** The addition to our information for research participants

For participants who are “going public^a^”
If you are one of the minority of participants in our study who is also talking to the media, running a Facebook campaign, writing an Internet blog, or tweeting about your experience (or if you might do so in future), please read this.
*How we will anonymize your interview*
We will try to ensure that nobody can identify you from extracts from your interview in the following ways:
1. We will change your name and the names of people in your family, other personal contacts, and the names of the professionals who cared for your loved one.
2. We will change the names of any hospitals, residential care homes, or rehabilitation units that you mention—and the names of towns and cities where they are located and/or where you live.
3. Unless you specifically give consent to the contrary, we will modify your occupation (if you mention it) and that of the person with brain injury to make you less identifiable.
4. We will alter or remove any other details you request. We can also remove some extracts from your interview and assign them a different pseudonym and identification number so that they cannot be identified as having been spoken by the same person.
However carefully we anonymize your interview, though, if you are “going public” about your family’s experience (e.g., talking to journalists, using social media), it might be possible for someone who has read your story in these other contexts to identify you when they read extracts from the interview you did with us. For example, this might happen if you have an unusual story with distinctive features that people will recognize, if you use very similar words and phrases in both contexts, or if you choose to illustrate your Facebook page with an image that you have also given to us to use in the Postcard Project.^b^
We hope you understand that if you are “going public” in other contexts we cannot guarantee your complete confidentiality. There is always the possibility that someone who has read about you in another context might then be able to identify you from the interview extracts. Your participation in our research is on the basis that you understand and accept the risk of being cross-identified in this way.

^a^We have emphasized throughout this article that those posting online do not necessarily see themselves as “going public” and may still maintain strong expectations of privacy; however, the phrase “going public” was used in the consent form as a catch-all phrase for convenience and so as not to complicate issues for the participants when reading it.

^b^The Postcard Project was an initiative in which family members chose or produced pictures to express something about their experience and wrote short messages on a postcard for display as part of an exhibition linked to the research.

We thus made participants aware that while we would do everything we could to keep them anonymous, these efforts could be undermined if their case had received a lot of media attention. We also highlighted to them the possibility of their words being recognized from media interviews they may have given, as well as from information posted on social networking sites, something that they may not have considered when agreeing to participate in the research. Developing a strategy to make interviewees aware of these issues prior to their participation at least gave them the opportunity to opt out if they were uncomfortable with this risk of identification or highlight parts of what they said to us that they would like dissociated from other parts of the interview. Additionally, throughout the research process the interviewers became more skilled in identifying anonymity issues at points when they arose in interviews. They were therefore able to discuss these issues with the interviewee, and either devise a way of disguising the potentially identifying section, or at least make the interviewee aware of the risk posed to their anonymity.

## Conclusion

In this article we have outlined the challenges that online information can pose to research participant anonymity, focusing on the online availability of three types of sources: media reports, court transcripts, and participants’ use of social media. While our study may be unique in encountering challenges relating to all three areas, the issues discussed will be relevant to many researchers working with qualitative data. We outlined the ways in which we attempted to tackle some of these issues, including:

- Devising elaborate strategies for disguise involving altering non-essential details.
- Presenting extracts from the same participant under multiple pseudonyms.
- Collaborating with participants over anonymity issues during the interview itself.
- Being very explicit about the extent of the anonymity we could offer, both before the interview in writing and at the end of the interview in person.
- Amending our consent form to explicitly make participants aware of the potential for readers to cross-link online information with interview data (see Saunders et al. 2014 for further discussion of these strategies).

Our discussion gives rise to some general ethical issues. First, the accessibility of online information has implications for the consent process, as consideration must be given to the increasing range of ways in which participants can be identified in research findings. As van den Hoonaard and van den Hoonaard (2013, p. 33) artfully put it, “anonymity is like a watertight barrel held underwater. The pressures that lead to holes in the researcher’s efforts to maintain anonymity are always there.” To extend their metaphor, we would argue that the potential for cross-linking information online means that this anonymity barrel may be close to bursting point. The importance of making participants aware of the limits to anonymity despite the researcher’s best efforts (Walford 2005) is thus even more pressing in the Internet age because participants may not consider the link that could be made between their posting of information online and what they said in a one-off interview, which could result in them experiencing future harm.

The issue of future harm is one that relates to the ethical treatment of human subjects more generally, as often harm cannot be reliably anticipated at the time when consent is sought (Wiles et al. 2012, p. 47); therefore, participants may be unhappy with what they originally consented to, or alternatively they may have originally derived a different perception of the consent process than that which the researcher intended (van den Hoonaard & van den Hoonaard 2013). We highlight the possibility that this unanticipated future harm could result from information posted online *subsequent* to research publication, either as a result of families going to court, giving media interviews or future social networking. This puts researchers in an almost impossible position of having to decide what information is safe to leave in and what could become identifying in the future. Therefore, it is imperative that the researcher makes this risk very clear to participants in the consent form, as well as reiterating this in each interview, as we attempted to do in the additional information provided to participants both verbally and in writing (see Table 1 and extract presented above).

Previous research has suggested various methods of collaborating with participants over consent issues; for instance, offering participants a “right to reply” to interpretations with which they do not agree (Walford 2005) or returning to participants for consent once there is a clearer idea of how data will be used (Kaiser 2009). We took the latter approach with some interviewees where anonymity was a particular concern, for example, returning a transcript to an interviewee once we knew which extracts we would like to use and inviting her to delete bits. Our discussion of researcher-participant collaboration here relates to participants having input about how they are represented in terms of anonymity issues, and having the opportunity at various points to suggest deletions or alterations to information that they deem potentially identifying. We are not talking here about participants responding to analytic interpretations of what they have said, as discussed by Walford (2005). This researcher-participant collaboration vis-à-vis anonymity issues was crucial, for example, where an interviewee wanted to remain unidentifiable to members of her own family (this was a task only she could have done, we could not have anticipated the fragments which might have made a family member recognize the speaker). We also collaborated with participants within the interview itself (as in the rubber gloves example). Although such approaches may not be practical in some research contexts, it has advantages in allowing researcher-participant negotiation at the time of the initial data-collection or at the time of initial analysis. This allows for a case-by-case, casuistic approach (Markham & Buchanan 2012) to informed consent, as there may be specific issues related to particular cases that are unforeseen when writing a consent form and that cannot be generalized to the rest of the data set.

The issues we have discussed vis-à-vis informed consent and participants’ privacy have been given significant attention in a recent report by the U.S. National Research Council (NRC) (2014), which outlines a range of recommendations about human subject research. In line with the arguments we have presented in this article, the U.S. NRC emphasizes the ongoing nature of the consent process, and the necessity for researchers to at times make situational judgments about consent issues:

Some types of social and behavioral research … involve research where the researcher cannot predict how the research will evolve and may not even be able to identify all participants until some data have been collected … In that case, the researcher should apply good professional judgment to tailor informed consent to the situation at hand. (2014, p. 92)

That the U.S. NRC affords such salience to these issues supports our argument that the broader ethical issues addressed in this article are not relevant only to a small pool of qualitative researchers, but rather they are being debated on the larger international stage and are considerations vital to anyone conducting human subject research. This article is well placed to contribute to this discussion—the challenges we have highlighted regarding the relationship between online information and interview data and the implications this has for thinking about privacy, and the novel ways we suggest to collaborate with interviewees, as well as the practical illustration of these methods—have the potential to both contribute to, and extend, the discussion of anonymity and informed consent.

Our anonymizing work aimed to strike a balance between participant protection and maintaining the integrity of the data, and we advocate a considered, case-by-case approach to anonymizing, which may include discussing specific issues with ethics boards and Institutional Review Boards where relevant. Having shared some of the challenges we have faced and the solutions we came up with, we would encourage other researchers to also present practical illustrations of the challenges they have encountered in order to open up a discussion about how to manage anonymity issues in the face of the increasing accessibility of online information. It could well be that in the contemporary, information saturated society in which we live, complete research participant anonymity is indeed a thing of the past (in so far as it has ever truly been possible to achieve). This does not render qualitative researchers entirely powerless, but what it does mean is that they need to be even more innovative and resourceful in coming up with ways to minimize the risk of participant identification where possible.
